# A Socio-Ecological Framework for Cancer Prevention in Low and Middle-Income Countries

**DOI:** 10.3389/fpubh.2022.884678

**Published:** 2022-05-26

**Authors:** Tomi Akinyemiju, Kemi Ogunsina, Anjali Gupta, Iris Liu, Dejana Braithwaite, Robert A. Hiatt

**Affiliations:** ^1^Department of Population Health Sciences, Duke University School of Medicine, Durham, NC, United States; ^2^Duke Cancer Institute, Durham, NC, United States; ^3^Department of Public Health Sciences, University of Miller School of Medicine, Miami, FL, United States; ^4^Department of Epidemiology, University of Florida, Gainesville, FL, United States; ^5^University of Florida Cancer Center, Gainesville, FL, United States; ^6^Department of Epidemiology and Biostatistics, University of California, San Francisco, San Francisco, CA, United States; ^7^UCSF Helen Diller Family Comprehensive Cancer Center, San Francisco, CA, United States

**Keywords:** cancer prevention, LMIC, low-income countries, middle income countries, socio-ecological framework

## Abstract

Cancer incidence and mortality rates continue to rise globally, a trend mostly driven by preventable cancers occurring in low-and middle-income countries (LMICs). There is growing concern that many LMICs are ill-equipped to cope with markedly increased burden of cancer due to lack of comprehensive cancer control programs that incorporate primary, secondary, and tertiary prevention strategies. Notably, few countries have allocated budgets to implement such programs. In this review, we utilize a socio-ecological framework to summarize primary (risk reduction), secondary (early detection), and tertiary (treatment and survivorship) strategies to reduce the cancer burden in these countries across the individual, organizational, community, and policy levels. We highlight strategies that center on promoting health behaviors and reducing cancer risk, including diet, tobacco, alcohol, and vaccine uptake, approaches to promote routine cancer screenings, and policies to support comprehensive cancer treatment. Consistent with goals promulgated by the United Nations General Assembly on Noncommunicable Disease Prevention and Control, our review supports the development and implementation of sustainable national comprehensive cancer control plans in partnership with local communities to enhance cultural relevance and adoption, incorporating strategies across the socio-ecological framework. Such a concerted commitment will be necessary to curtail the rising cancer and chronic disease burden in LMICs.

## Introduction

Cancer is a leading cause of death globally, despite significant progress toward prevention and improved cure rates for some cancers (1). An estimated 19.3 million new cancer cases and 10 million cancer deaths occurred in 2020 (1). In 2017, while 51% of cancer incidence occurred in countries of high socio-demographic index, these countries accounted for only 30% of global cancer deaths and 24% of cancer disability-adjusted life-years (DALYs) (2). Notably, the global burden of cancer is estimated to increase to 28 million cases by 2040, a 47% increase from 2020, driven largely by increasing cancer incidence in low and middle-income countries (LMICs) (1). This trend is attributable to the epidemiologic and demographic transitions, globalization and associated changes in lifestyle factors (1), and marked geographic variations in access to cancer prevention and care.

**Figure 1:**
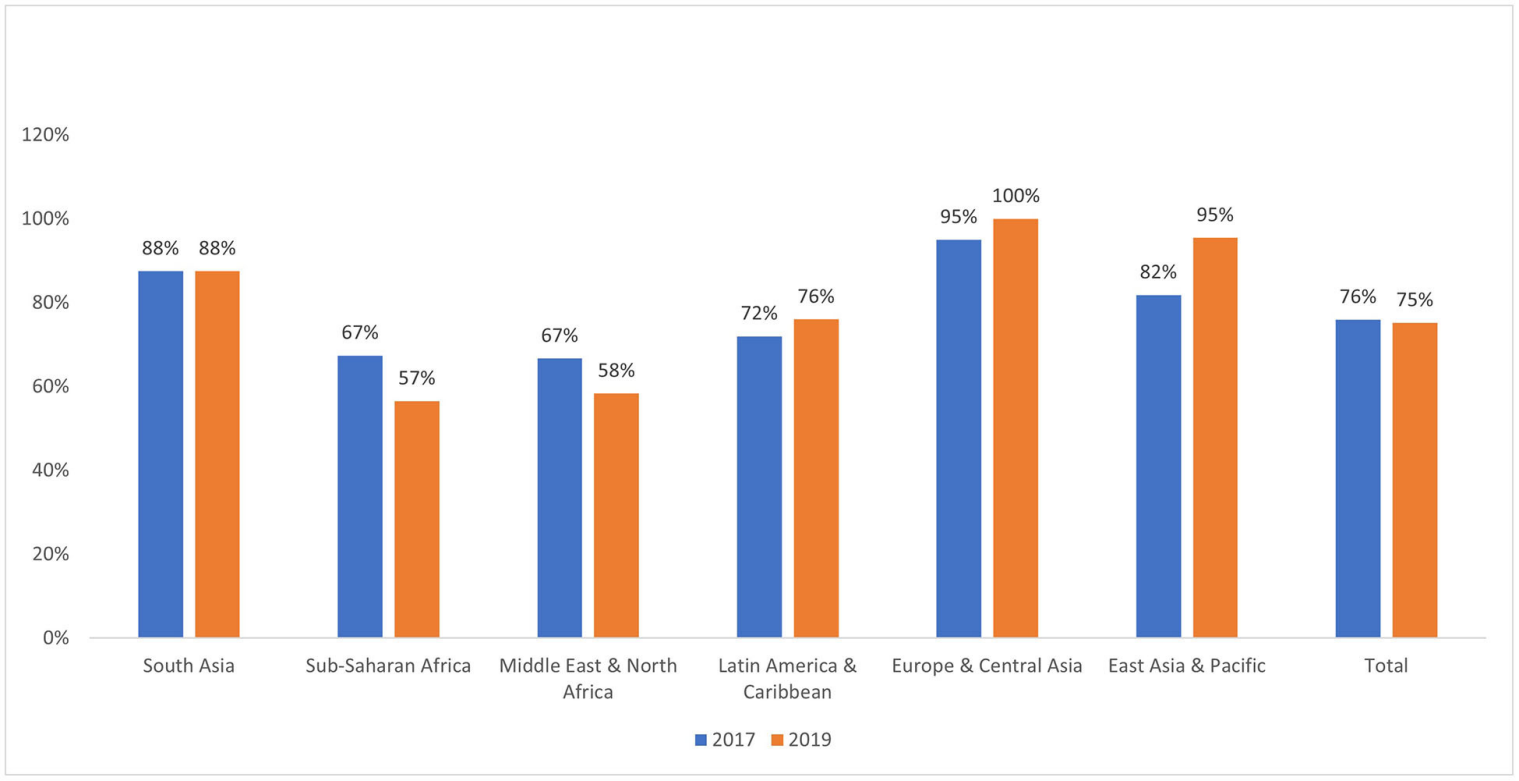
Percentage of LMICs with an operational policy/plan for cancer in years 2017 and 2019.

In 2011, a high-level meeting of the United Nations (UN) General Assembly on Noncommunicable Disease Prevention and Control developed a plan to address cancer's role in the global health agenda, alongside other noncommunicable diseases (3). Their report highlighted growing concerns about the rising burden of cancer in LMICs, cautioning that many LMICs will be ill-equipped to cope with the projected increase in the number of patients with cancer without well-funded, comprehensive cancer control plans (4, 5). Some progress has been made since the UN General Assembly meeting; for instance, in 2017, 101 out of 133 LMICs had an operational policy/strategic action plan for cancer prevention/treatment, though few made specific financial commitments to implement such plans (6, 7). By 2019, this number had stagnated at 100 out of 133 LMICs, with decreases in the Middle East & North Africa and Sub-Saharan Africa regions (Figure 1). Presently, many LMICs still lack funded, comprehensive cancer control programs that incorporate primary, secondary and tertiary prevention strategies across the multiple levels of action: individual, community, health system and government/national policy (8).

Intervention strategies that emphasize risk reduction are crucial in the fight against cancer. These strategies can potentially reverse the projected increases in cancer rates and thus reduce the burden on fragile health systems in LMICs. Many cancer-related risk factors, such as obesity and smoking, are also associated with other non-communicable diseases, including diabetes, COPD and cardiovascular disease; therefore, reducing the prevalence of these major risk factors may help to reduce the burden of cancer as well as other chronic diseases, making it a cost-effective strategy for national health policy (9). However, lack of rigorous evaluation is a major challenge in many LMICs, complicating efforts to develop population-specific cancer prevention strategies. Empirical data on the burden of known cancer risk factors such as tobacco/smoking, infections, occupational exposures, excess alcohol use, unhealthy diet/nutrition, and lack of physical activity can provide insights for areas of primary prevention strategy emphasis. Among the main causes of cancer, tobacco had the largest impact in both LMICs and high-income countries (HICs) in 2012 (10), however there are geographic variations in the trends for this and other major cancer risk factors (Table 1), highlighting a need for country-specific analysis and evaluation.

**Table 1:** Impact and trends of some of the major causes of cancer in LMICs and HICs.

**Risk Factors**	**LMICs**	**HICs**
Tobacco	+++↑	+++↓
Infection	+++↓	+↓
Poor nutrition	+↑	++↑
Alcohol use	+↑	+↓
Occupational exposures	+↑	+↓
Hormones	+↑	++↑
Radiation	+↔	+↔

*Adapted from Braithwaite et al. (10)*.

*Plus signs indicate the strength of impact of each risk factor on cancer incidence; arrows indicate trends in the prevalence of each risk factor*.

Though screening programs for breast, cervical and oral cancers exist in many LMICs, these programs are often localized in specialized clinics, mostly in urban areas, and difficult to access due to administrative, financial, and geographic barriers (11). Oncology specialists in many regions are few relative to population size, and often impeded in their effectiveness by lack of access to effective cancer medication and surgical tools (12). Cancer screening and treatment opportunities that do exist in LMICs are often unorganized, fragmented and operate in silos, creating barriers for patients who may lack the knowledge or financial resources to access or navigate the steps between screening, follow-up, diagnosis, and treatment (5, 12). In addition to efforts to develop and promote effective cancer screening in LMICs, strategies to enhance timely diagnosis, quality cancer treatment and follow-up would be beneficial. For meaningful progress to be made toward cancer prevention in LMICs, a comprehensive cancer control strategy, co-developed with various constituents, ranging from individuals to advocacy groups/organizations, and communities, is needed. If not already in place, countries will benefit from developing and disseminating, with input from all relevant stakeholders, a comprehensive cancer control plan coupled with sustained long-term funding to implement the strategies proposed in the plan.

## Actionable Recommendations

In the following sections, we describe a range of evidence-based cancer prevention strategies that can be facilitated by individuals, organizations, communities, and governments in LMICs. This article presents a cohesive and action-oriented summary of cancer prevention strategies, incorporating the WHO “Best Buys” (9), using a socio-ecological model (SEM) framework. SEM is a conceptual framework first introduced in the late 1970's (13, 14) to enhance strategic effectiveness across multiple levels (15–18). Similar approaches have been adapted by the Centers for Disease Control and Prevention's Colorectal Cancer Control Program (19), and in studies evaluating health disparities and uptake of health behaviors (20, 21). Here, we build on the WHO's *Global Action Plan for the Prevention and Control of Noncommunicable Diseases* (22) to center cancer prevention strategies at the individual, community, organization and government levels. Examples of these strategies are shown in Table 2. A comprehensive, evidence-based strategy for cancer prevention, with sustainable infrastructure and rigorous evaluation, will be key to reducing the burden of cancer in LMICs in the next decade.

**Table 2:** A socio-ecological model for cancer prevention in low and middle-income countries.

	**Primary Prevention: Risk Reduction**	**Secondary Prevention:** **Screening and Detection**	**Tertiary Prevention: Treatment, Follow-up, Palliative care**
• Develop and disseminate a population-specific, comprehensive cancer control plan with input from key stakeholders			
• Provide adequate and sustained funding to support specified plan, including surveillance, research, and implementation			
Government/ public policy	• Fund research to evaluate and reduce the prevalence of known cancer risk factors, specifically smoking, alcohol, obesity, cancer-associated infections, and environmental/occupational exposures • Promote the integration of primary prevention of cancer (risk factor reduction) within routine primary health care • Develop and enforce national policy on nutritional standards, food and water quality, environmental exposures and cigarette tax/indoor smoking bans	• Develop and evaluate national capacity for low-cost breast, cervical and colorectal cancer screening • Develop and disseminate national cancer screening guidelines to stakeholders including insurance companies, hospitals, medical organizations and individuals • Create programs for free/subsidized cancer screening for low-income individuals • Promote integration of routine cancer screening within healthcare systems	• Develop, disseminate and evaluate national guidelines for standardized stage-appropriate treatment for each cancer type • Provide training and funding opportunities for continuing medical education in oncology and palliative care for medical professionals • Negotiate for reduced-cost therapeutic agents and equipment for cancer treatment, including narcotics for pain management, and promote equitable distribution of drugs and equipment nationally • Provide funding to subsidize cancer treatment cost for low-income individuals
Community	• Promote awareness of cancer as a chronic disease, and disseminate information on the role of risk factors in cancer development • Create community resources (e.g., farmer's markets for fresh, affordable healthy food options), and advocate against unhealthy fast-food options • Create and advocate for green spaces to promote outdoor physical activity	• Promote awareness of routine cancer screening and early diagnosis as key for cancer survival, and create a culture of routine check-ups for early symptoms of cancer (e.g., suspicious breast lumps) • Advocate for accessible and affordable cancer screening and trained healthcare providers to provide routine and diagnostic screening and referrals	• Reduce stigma associated with cancer diagnosis by increasing knowledge of cancer as a chronic health condition •Promote instrumental (transportation, medication reminders) and emotional (having a confidant) support for cancer patients • Conduct fundraisers to provide resources to fund research, support for patients and advocacy
Organization	• Promote healthy food, physical activity, reduced environmental and occupational exposures and HPV vaccination in schools and workplaces • Promote free or reduced cost smoking cessation programs for low-income individuals • Advocate for policies to eliminate harmful chemicals in the air, water, and food, and for cigarette taxes and/or indoor smoking bans	• Develop cancer screening awareness programs and provide/subsidize cancer screening services for the underserved • Develop fundraising campaigns to address individual barriers to cancer screening (e.g., transportation, screening, and follow-up referral costs)	• Provide medical leave policies and generous insurance policies for cancer patients and for immediate family members to care for patients • Advocate for ongoing healthcare provider training on culturally appropriate handling of cancer treatment and end-of-life care
Individual	• Reduce/eliminate known cancer risk behaviors such as obesity, poor nutrition, low physical activity, smoking, excess alcohol; increase breastfeeding and HPV vaccine uptake • Increase awareness of non-modifiable risk factors: family history, genetic mutations	• Obtain regular age-appropriate, breast, cervical and colorectal cancer screening	• Follow healthcare provider treatment regimen closely • Seek the help and support of family members
Outcomes	Reduce cancer incidence	Reduce late-stage disease	Reduce mortality

### Primary Prevention of Cancer in LMICs

Primary prevention strategies for cancer aim to reduce the prevalence of known cancer risk factors such as smoking, cancer-associated infections, and other lifestyle related risk factors. An estimated 1.3 billion people smoke worldwide, 80% of whom reside in LMICs (23). Tobacco smoking is estimated to be responsible for about 21% of cancer deaths globally, and about 18% of cancer deaths in LMICs (24). Tobacco is an established risk factor for cancers of the lung, head and neck (oral cavity, pharynx, and larynx), nasopharynx, esophagus, stomach, pancreas, liver, kidney, bladder, cervix and blood (leukemia) (24). For example, cigarette smoking is known to increase the risk of lung cancer by 15–30-fold, and laryngeal cancer by 10-fold (24). Furthermore, in LMICs, cancer-related infections are responsible for a significant proportion of cancer cases. In 2008, infections accounted for 23% of new cancer cases in less developed countries, compared with 7% of new cases in more developed countries (25, 26). *Helicobacter pylori* is known to colonize the gut of approximately 50% of the world's population and is a major risk factor for gastric cancer (27, 28). Human papillomavirus (HPV) is a causal risk factor for cervical cancer (29), which was responsible for over 300,000 deaths globally in 2018, with over 80% of these deaths occurring in LMICs (30). Hepatitis B and C are responsible for 56% and 20% of liver cancer cases worldwide, respectively (26). Other modifiable factors associated with increased cancer risk include excessive alcohol consumption, obesity, unhealthy diets, and lack of physical activity (31–34). Furthermore, common environmental risk factors in LMICs include indoor toxic air pollutants from cooking, excessive sun exposure, outdoor air pollution, and occupational exposure to carcinogenic agents (32, 33).

#### Individual Level

To prevent deaths from smoking-related cancers in LMICs, more intense efforts to reduce the prevalence of smoking will be necessary (23). An analysis of data from 43,540 participants in the Global Adult Tobacco Survey in 14 LMICs showed that approximately 82%, 14%, and 4%, respectively, of smokers were in precontemplation, contemplation, and preparation stages of smoking cessation based on the transtheoretical model (35). Smoke-free policies and exposure to anti-smoking messages were associated with increased odds of contemplation and preparation to quit smoking (35). Educating adolescents about the dangers of smoking in schools was found to be most effective in reducing susceptibility to smoking among Malaysian females (36). Increased knowledge of smoking harm and higher perceived health risk of smoking were associated with reduced smoking susceptibility among Thai females and Malaysian male adolescents (36). In China, an online game-based 22-day marketing campaign against tobacco was found to influence attitudes toward smoking; 57 vs. 73% of participants indicated negative attitudes toward smoking before vs. after the campaign (37). At an individual level, efforts can be made to advocate for smoke-free policies and promote anti-smoking education among adolescents. Vaccinations for HPV have been found to be successful in preventing 70% of cervical cancers in adequately vaccinated populations (38). Novaes et al. showed that introducing the HPV vaccine to a cohort of 11-year-old children through the National Immunization Program in Brazil will result in an estimated 229 deaths avoided and 6,677 disability-adjusted life years (DALYs) averted in the vaccinated cohort across their lifetime (39). Additionally, maintaining a healthy weight, routine physical activity, prolonged breastfeeding, safe sexual practices, and use of sunscreen or limiting sun exposure are also known to reduce the risk of cancer (32, 33, 40–43). Increased consumption of fruits and vegetables is beneficial in preventing deaths from cancers (44–46). The promotion of sustainable strategies to reduce smoking, promote uptake of HPV vaccines, and integrate healthy dietary practices as part of a healthy lifestyle for individuals and families may help to reduce the incidence of cancer in LMICs.

#### Organizational Level

Large-scale vaccination programs at the organizational level have proven feasible and effective. For example, a school-based HPV vaccination program among 10–16-year-old adolescents in Brazil achieved 97% three-dose vaccination uptake and completion, demonstrating the feasibility of implementing large-scale HPV vaccination programs in LMICs (47). Additionally, the Gardasil Access Program, which provided access to vaccination against HPV at no cost, was conducted in 14 LMICs. The mean vaccine uptake rate was 89% and the vaccine adherence rate was 91%. Vaccination in schools was significantly associated with vaccine uptake rates (48). Furthermore, Verstraeten et al. conducted a systematic review to evaluate the effectiveness of school-based obesity interventions in LMICs (49). They found that interventions that positively changed both proximal (i.e., diet or physical activity) and distal (i.e., BMI or overweight/obesity prevalence) outcomes were generally multicomponent, education-based interventions that provided physical activity sessions or classes that covered healthy foods and nutrition (49). In the context of workplace interventions to address occupational hazards, a targeted public education program among pesticide handlers in two villages in South India was associated with significant improvements in workers' knowledge and practice regarding hazardous chemical safety and handling (50). This intervention specifically targeted a region with a high prevalence of occupation-related pesticide poisoning. Workplace interventions to address smoking may also be beneficial. For example, a randomized controlled trial of large industrial workplaces in Thailand found high acceptance of monetary incentive programs for promoting smoking abstinence, with programs that offered $40 individual bonuses associated with increased long-term abstinence compared with usual care (51). Cancer prevention strategies integrated within school and work settings can be effective in reducing risk factors associated with cancer and can be feasibly integrated into organizational practice.

#### Community Level

Community-based participatory research (CBPR) methods engage local communities through equitable, partnered efforts, and may offer immense potential for cancer prevention programs (52, 53). Such an approach was utilized in a mother-child screen/treat and vaccinate program in Peru. Community health workers first completed a 3-day educational training session, after which they modified the program to suit their individual communities. The program involved two key components: HPV self-sampling and cryotherapy to screen/treat mothers, and Gardasil to vaccinate their children. The community workers registered participants via a door-to-door approach. Results showed that 97% of participants provided HPV test samples, 94% of those who were HPV positive were treated, and over 90% of girls registered received their 1st and 2nd doses of the vaccine (52). Additionally, a study of the Gardasil Access Program conducted in 14 LMICs found that community involvement during follow-up was significantly associated with the vaccine uptake rate among girls (48). CBPR methods have also proven effective beyond vaccinations. For example, in Iran, a study conducted to increase physical activity among women used the CBPR approach and was found to be effective; the percentage of women who reported participating in physical activity increased from 3% at baseline to 13% in the intervention group, while there was no change from baseline in the non-intervention groups (54). CBPR methods can be particularly beneficial in leveraging existing traditions and cultures in the process of disseminating cancer prevention information. For instance, in Zambia, the use of traditional marriage counselors was adopted to create awareness about cervical cancer. The counselors received training regarding screening, vaccination, early signs and symptoms, and the harmful effects of practices such as the use of vaginal herbs that can disrupt the normal vaginal flora and increase the transmission of HPV (55). Additionally, communities can further leverage political capital to advocate for shared resources including parks and green spaces to enhance physical activity, and zoning laws to promote the availability of fresh produce and limit the propagation of unhealthy food options, liquor stores or access to cigarettes. Community-based methods may also be beneficial for identifying and addressing context-specific barriers to cancer prevention, such as lack of spousal or family support, lack of awareness on reproductive health issues, religious beliefs, and stigma associated with discussing reproductive health (56, 57).

#### Policy Level

At the policy level, an essential first step to cancer prevention may be addressing efforts by tobacco companies to undermine effective tobacco control (58). Article 5.3 of the World Health Organization's Framework Convention on Tobacco Control offers governments a set of strategies to protect public health against commercial and other vested interests of tobacco companies (58). The WHO recommends cost effective and feasible interventions to reduce smoking in LMICs such as increased taxes and prices on tobacco products, indoor and public smoking bans, age-limits on purchases of tobacco products, bans on tobacco advertising, promotion and sponsorship, and health warnings on all tobacco packages (9). In a study of such policies adopted from 2007 to 2010 in 40 countries, including many LMICs, Levi et al. estimated that their implementation would result in 15 million fewer smokers and 7.4 million premature deaths averted by 2050 (59). In Vietnam, the country with the highest male smoking rates in the world (72.8–74.3%) in the 1990's, there were dramatic declines in national rates of lung cancer following the implementation of the National Tobacco Control Program (NTCP) (60). Implementing tobacco control policies may be challenging in countries that are financially invested in the tobacco industry, however governments must weigh short-term financial benefit against long-term catastrophic health expenditure due to increasing cancer burden. The WHO also recommends cost-effective policies to address alcohol consumption, including excise taxes on alcoholic beverages, bans or comprehensive restrictions on advertising, and restrictions on the physical availability of retailed alcohol (9). Brief psychosocial intervention for individuals with hazardous alcohol use may also be beneficial (9). In the context of improving diet, the WHO recommends policies focused on reduced salt intake via the reformulation of food products to reduce salt content in foods and meals (9). Zoning laws to reduce the expansion of unhealthy food options to dense urban areas or low-income neighborhoods, and subsidies to increase availability and affordability of fruits and vegetables, may also help to improve diet quality and reduce the risk of cancer and other non-communicable diseases in LMICs (61). To address infection-related cancers, implementation of HPV vaccination policies can be accomplished in LMICs through political action, and financial resources for cervical cancer prevention in school and clinic settings (48, 62). Policies ensuring universal access to subsidized age-appropriate Hepatitis B and HPV vaccinations are recommended as part of national immunization programs (63). Additionally, food safety and hygiene programs to address sewage contaminated food and water exposure may reduce *H. pylori* infection and gastric cancer risk, and rigorous environmental health standards can reduce emissions that contribute to toxic air and water pollution (64). Furthermore, policies that require the incorporation of cancer prevention recommendations into routine healthcare practice can be successful when appropriately implemented. Evidence suggests that healthcare workers can influence health behavior changes by endorsing prescriptions for lifestyle changes, such as increased fruit and vegetable intake, increased exercise, and vaccination uptake (65). At the government level, policies that prioritize and support cancer risk reduction strategies can be extremely powerful in catalyzing local and regional cancer control activities.

### Secondary Prevention of Cancer in LMICs

Secondary prevention of cancer is primarily focused on screening and detection efforts to detect cancer early. Cancer screening is an effective approach for detecting breast, cervical, lung, and colorectal cancer (66). Effective screening efforts are associated with earlier stages at diagnosis, and detection of cancers that are more amenable to curative treatment, leading to better outcomes (67). There are several barriers to the secondary prevention of cancer in LMICs including limited infrastructure, few trained personnel, high cost of equipment purchase and maintenance, and inadequate training of healthcare providers (68, 69). In areas where screening programs exist, some LMICs experience a simultaneous over- and under- utilization of these services; screening resources and personnel are often clustered in urban settings, where there is a large population base and high demand, limiting opportunities for screening in rural settings. Even among regions where screening programs have been found to be effective, there are challenges in scaling up these interventions. Therefore, to increase utilization of routine cancer screening in LMICs, strategies to provide reliable and affordable screening equipment, support a well-trained screening workforce, and reduce loss to follow-up between screening, diagnosis, and treatment, particularly among rural populations, would be beneficial (69). Improving awareness of the importance of screening in early detection and prevention of cancer is a critical first step toward the secondary prevention of cancers in LMICs, followed by investments in high-quality screening programs integrated into routine healthcare.

#### Individual Level

Several screening programs have proven effective in LMICs at the individual level. For example, programs involving visual inspection with acetic acid (VIA), HPV-based screening, or single-visit screen/treat with cryotherapy or thermo-coagulation have been found to prevent neoplasia and cervical cancer deaths in clinical trials conducted in LMICs by the International Agency for Research on Cancer (IARC) (70). Additionally, review articles of studies from LMICs indicate that HPV self-sampling by women is also useful as a primary screening strategy (71, 72), especially since this method can overcome cultural barriers such as discomfort with the pap smear procedure, fear of medical procedures, and unwillingness to be examined by male physicians (73). For breast cancer, the WHO recommends mammography screening for women ages 50–69 years in LMICs, only where there is a fairly strong health system and shared decision-making strategies for women and providers (74). Although there is generally limited data on the cost-effectiveness of mammography screening in LMICs, IARC suggests that mammography may also be effective for women ages 70–74 years. Furthermore, IARC recommends that in resource limited settings, clinical breast examination is potentially beneficial and may help shift the distribution of breast cancer to lower stages at diagnosis (75). Additionally, the Breast Health Global Initiative (BHGI) recommends diagnostic breast ultrasound scans in low resource setting where mammograms are not available for high-risk women (76). For example, a study conducted in a tertiary hospital in Nigeria found ultrasound scans to be effective for breast cancer screening and prevention in rural settings with low resources (77).

#### Organizational Level

At the organization level, strategies promoting cancer screening awareness and utilization may include annual health training programs for staff in workplaces to encourage screening and provide information on how to access screening. In LMICs, worksite screening interventions have also been found to be effective. In a study conducted in Malaysia, an intervention group was provided with worksite cervical cancer screening, while a control group received usual care from existing cervical cancer screening programs. The uptake of cervical screening was twice as high in the intervention group compared to the control group, highlighting that cancer screening utilization can potentially be increased by interventions in the workplace (78). In another example, in Nanjing China, a workplace-based intervention for breast cancer included breast cancer education and screening navigation. Evaluation of this intervention found dramatic increases in the uptake of mammography from 10% at baseline to 73% at 6-month follow-up (79). Organizations may also improve utilization of cancer screening by including free or low-cost screening as part of health insurance plans and providing time off from work to attend screening services on an annual basis. Additionally, workplaces may organize worksite screening programs as part of a holistic wellness approach; these provide comprehensive screening tests onsite that cover blood pressure, blood glucose, HIV tests, clinical breast exams and HPV tests. Examples of this approach can be seen in high-income countries such as the United States (US), where certain employers offer periodic screening as part of employee health benefits (80). Additionally, the US Centers for Disease Control and Prevention (CDC) promotes worksite prevention initiatives by providing resources for states to facilitate programs such as the National Healthy Worksite Program. This program involves several components, such as having a workplace health resource center among other health-promoting opportunities (81). If worksite interventions are coupled with a strong commitment to employee health privacy confidentiality, and goals geared toward improving health, these programs may prove to be cost-effective in the long-term for LMICs by reducing the burden of cancer and other non-communicable diseases in the workforce.

#### Community Level

There are several strategies that communities can deploy to ensure ample awareness and successful implementation of cancer screening programs, including peer coaching, community health fairs, and outreach programs. For instance, communities may advocate for resource-appropriate screening programs for cervical cancer using screen/treat approaches that involve VIA and excision of suspicious lesions. This approach has been shown to minimize loss to follow up and avoid delays in diagnosis and treatment (82). Programs providing VIA services in communities can also increase cervical cancer awareness through lay education, and by implementing the screen/treat approach via healthcare workers (health cadres, general practitioners, and midwives) trained in cryotherapy treatment for VIA positive individuals (83). In a large-scale randomized controlled trial conducted in rural Western Kenya, HPV self-sampling implemented within community health campaigns has proven effective for cervical cancer screening, with greater reach than screening in health clinics (84). Cost analyses indicated that screening within community health campaigns was cheaper per woman compared to screening in clinics ($25.00 vs. $29.56), suggesting that integration of cervical cancer self-screening activities into community health campaigns may offer a viable low-cost strategy (85). Communities can also collectively invest in mobile units to improve outreach to rural or hard-to-reach areas and facilitate the linkage of patients with suspicious lesions to regional health clinics, though these approaches may be expensive and require government support and investment. Several studies have shown that mobile units are effective in delivering cervical (86) and breast cancer screening in hard-to-reach areas (87). Other community activities that may enhance awareness and increase utilization of cancer screening include the use of radio broadcast and car loudspeakers to advertise the timing and location of screening services, as well as the use of home visits by community health care workers (87).

#### Policy Level

National, state, and local governments in LMICs are critical to developing policies that support the implementation of effective, feasible, affordable, and sustainable cancer screening programs. Cancer control policies for screening require sustained funding, continuous training of health personnel (specialists, nurses and community health workers), monitoring and evaluation of new and existing programs, and assessment of geographic coverage of screening services (63, 69). Very few LMICs have developed nationwide or state-wide cancer screening programs, despite evidence that these are highly effective toward early detection of cancer and reduced mortality (88, 89). Examples of LMICs that have scaled up evidence-based population screening programs for cancer prevention and control include Zambia, Bangladesh, Guatemala, Honduras, and Nicaragua (90), all countries that managed to implement WHO-recommended services despite budgetary constraints (90). In Bangladesh, a National Cancer Control Strategy and Action Plan was developed with the aim of delivering quality and timely services that included health promotion, early detection, and vaccination (91). This program has been initiated for breast and cervical cancer (91). In countries where nationwide screening programs may not be feasible, a sustained commitment of resources to these programs may start in specified regions and spread to other parts of the country as more resources become available. However, nationwide screening programs are not without limitations. For example, the Guatemalan Ministry of Health worked with non-governmental organizations (NGOs) to adopt the VIA as a low-cost alternative to pap smears for cervical cancer screening (92). Programs included cervical cancer screening campaigns, prevention conferences, and VIA training courses. There were several challenges to scaling-up these interventions, including high staff turnover, and concerns over training quality and problems with cryotherapy referrals when immediate treatment for VIA-positive women was unavailable (92). LMICs looking to expand population-based screening services can learn from past obstacles and challenges experienced by countries that have already attempted such programs, and strategies such as peer-country mentorship may go a long way in helping more countries develop robust secondary prevention programs. Additionally, governmental funding to support implementation and dissemination research to adapt evidence-based interventions for the local context, and to identify effective implementation strategies given local constraints can significantly enhance the success and sustainability of screening programs.

### Tertiary Prevention of Cancer in LMICs

Tertiary prevention of cancer primarily centers on treatment, follow-up, and palliative care efforts to reduce morbidity and mortality and optimize quality-of-life among cancer patients. Efforts that promote the adoption of a healthy lifestyle, access to treatment options, adherence to treatment regimens, and research on novel therapeutics and cancer outcomes within the country, may be highly beneficial in the tertiary prevention of cancer.

#### Individual Level

Just as in primary prevention, lifestyle factors have been found to play a pivotal role in the tertiary prevention of cancer (93, 94). Specifically, obesity, poor nutrition, physical inactivity, and continued smoking have repeatedly been shown to negatively impact outcomes among cancer survivors, including increased risk of cancer recurrence and mortality. For example, individuals diagnosed and treated for colorectal cancer, who attain a healthy weight by adopting an active lifestyle and healthy diet, are more likely to have better prognoses and improved outcomes (95). Overall mortality in this group has been reported to reduce by 40%, with significant improvements in quality of life during chemotherapy (95). Therefore, many of the aforementioned efforts to promote a healthy lifestyle may also be beneficial in the tertiary prevention of cancer. There are a number of other individual level factors that may improve cancer survivorship. McCutchan et al. conducted a systematic review of psychosocial influences on help-seeking behavior in LMICs (96). They found that the use of traditional, complementary and alternative medicine was a key barrier to medical help-seeking in LMICs, and was influenced by causal beliefs, cultural norms, and a preference to avoid biomedical treatment. They also noted that women face unique barriers, including needing family permission and experiencing stigma for cancer treatment (96). Culturally sensitive awareness campaigns and efforts to promote social, emotional, and tangible support by individuals, families, and communities for patients undergoing cancer treatment may improve survivorship in LMICs.

#### Organizational Level

Workplace prevention programs may be helpful in preventing the recurrence of cancer and its associated complications. As previously discussed, evidence exists that even after being diagnosed with cancer, maintenance of a healthy weight, proper diet and physical exercise are key activities in tertiary prevention (97). The American Cancer Society has provided guidelines on nutrition and physical activity for cancer survivors (97), which can be implemented in LMICs and promoted by workplaces in addition to health systems, oncologists, and physicians. Additionally, generous health insurance coverage and co-pay support through employment can substantially improve treatment utilization and adherence and reduce out of pocket costs and the financial burden of cancer. Effective workplace sick leave policies for cancer patients and immediate family members in care-taking roles may facilitate adherence to and completion of cancer treatment regimens by providing time and space for patients to attend to their health needs while promoting job retention and financial stability (98). During the COVID-19 pandemic, several countries temporarily strengthened paid sick leave programs, including El Salvador, Chile, Saudi Arabia, Trinidad and Tobago, and Uzbekistan (99). These efforts were instituted to reduce COVID-19 spread among workforces but offer a model for more sustainable, long-term sick leave policies that better support the needs of cancer patients in LMICs. NGOs and faith groups can also play a key supporting role in reducing the physical, financial and emotional toll of cancer diagnosis by providing tangible support, such as transportation, financial assistance, and caregiving, and emotional support for cancer patients.

#### Community Level

There are several activities that can be implemented at the community level to promote the tertiary prevention of cancer. Community efforts may be effective in reducing barriers to accessing and utilizing cancer treatment in LMICs. These efforts include both tangible support such as assistance with transportation for healthcare appointments/treatments and providing psychosocial support that counters fear, shame and stigma (96). In the context of practical concerns, communities can support instrumental needs such as transportation and childcare that are often barriers for patients to access care. In terms of psychosocial factors, stigma is often a consequence of low knowledge of the disease, so community efforts to raise awareness of cancer as a chronic illness may promote greater utilization of biomedical treatment. Beyond curative treatment, stigma also represents a major obstacle to high-quality palliative care in LMICs. For example, in parts of Africa, terminal illness is sometimes believed to be caused by the bearing of bad news, and in China, terminal illnesses may be regarded as resulting from some wrongdoing of the affected individual (100). Concurrently, there may be cultural taboos against open communication about death among physicians, hampering palliative care for terminal patients (100). Community interventions, such as mobile roadside clinics in Uganda and bereavement services provided by social workers, have been successful in addressing these barriers (100).

#### Policy Level

Based on extensive evidence delineating the importance of lifestyle modifications in both primary and tertiary cancer prevention, governments can invest in infrastructure that supports healthy diets and physical activity for citizens. Examples of such programs are described above in the discussion of primary cancer prevention. Additionally, in the context of cancer treatment, it would be beneficial for governments to invest in capacity building by increasing the availability of high-quality cancer treatment facilities and upgrading existing facilities. Efforts to make surgical treatment, chemotherapy and radiation therapy available and affordable in LMICs would be helpful; currently, these treatment forms often exist only in capital cities, while in some countries, may not be available at all (101). For example, the use of radiotherapy is often insufficient in LMICs due to a lack of skills and equipment required to provide this treatment. Even where the equipment and skills are available, this is rarely enough to support patients who seek to attend a radiotherapy center from surrounding regions. Strategies to increase the availability of radiotherapy in LMICs would be beneficial. In line with this goal, the International Atomic Energy Agency (IAEA), made up of 138 UN member states, is focused on accelerating health by improving radiotherapy worldwide (102). Individual countries can increase support for workforce training and infrastructure capacity to hasten implementation. Furthermore, training of healthcare professionals for pain management and palliative care, which often involves radiotherapy, is crucial to ensuring effective pain control, distress management, and higher quality of life among cancer patients, but is often insufficient in LMICs. Training efforts must be adapted to the local context and consider cultural and practical implementation challenges. Several countries, such as Kenya, Tanzania, Uganda, and Zambia have developed and implemented national palliative care programs in collaboration with local universities, and others, such as Mexico have passed legislation mandating adequate training in palliative care for all healthcare personnel (100). Building on these existing examples, governments in LMICs can promote context-specific implementation research for palliative care. Additionally, governments in LMICs can play a key role in achieving access to morphine and other narcotics as part of comprehensive cancer pain management standards by passing relevant legislation; in Uganda, governmental collaboration and lobbying have achieved access to morphine and palliative care within the country's National Health Policy Plan and Strategy (103). Beyond addressing gaps in cancer treatment and palliative care, it would be beneficial for governments to invest in the development of cancer registries to document the prevalence and burden of cancer within their countries (63) and use these findings to inform activities for cancer prevention and control, and for routine evaluation of the impact of cancer control strategies in reducing the cancer burden (104).

## Discussion

In light of rapidly rising cancer incidence and mortality rates in LMICs and increased pressure on health systems in these countries, efforts to promote cancer prevention in LMICs are urgently needed. Here, we used the socio-ecological framework to summarize primary (risk reduction), secondary (early detection), and tertiary (treatment and survivorship) approaches to reduce the cancer burden across the individual, organizational, community, and policy levels. Following targets set by the global cancer community, it is crucial that governments develop and fund national cancer control programs (63). These programs will not only generate a roadmap for reducing the cancer burden but will also create avenues for collaborations with internal and external partners to increase technical and financial support to implement and sustain the cancer prevention strategy. Every LMIC can build on their existing strengths and resources, and partner with various stakeholders to co-develop and implement a cost-effective and sustainable national comprehensive cancer control plan, incorporating tactics across the socio-ecological framework. This concerted commitment is necessary to curtail the rising cancer and chronic disease burden in LMICs.

## Author Contributions

TA, DB, and RH: conceptualization. TA, KO, AG, IL, DB, and RH: writing–original draft and writing–review and editing. All authors contributed to the article and approved the submitted version.

## Conflict of Interest

The authors declare that the research was conducted in the absence of any commercial or financial relationships that could be construed as a potential conflict of interest.

## Publisher's Note

All claims expressed in this article are solely those of the authors and do not necessarily represent those of their affiliated organizations, or those of the publisher, the editors and the reviewers. Any product that may be evaluated in this article, or claim that may be made by its manufacturer, is not guaranteed or endorsed by the publisher.
